# Can a Reptile Live in the Stomach?

**Published:** 1849-01

**Authors:** M. C. Richardson

**Affiliations:** Hallowell, Me.


ARTICLE VIII.—*Can a Reptile live in the Stomach?*

*To the Editor of the Boston Medical and Surgical Journal*:—DEAR SIR,—Permit me to make the inquiry, through you, whether it is possible for a reptile to live in the human stomach? and, if so, how long? Could it not only *live*, but *grow* to some size there?

The reasons for making this inquiry are the following:—Mrs. W., who has usually enjoyed excellent health, has, during the summer past, been unable to attend to her ordinary business. Her appetite has been capricious. She has complained of a disagreeable sensation at the pit of the stomach, sometimes amounting to pain, and frequently attended with nausea. These symptoms increased in severity until, about a fortnight since, she ejected a live snake from her stomach. It was seven inches in length, and of the common green species. It lived two days in a bottle of water, and then died. I have it now preserved in spirits. Mrs. W. thinks she remembers having swallowed some object in a glass of spring water which she drank in the dark, in May or June. She has now recovered her usual health. Yours, &c.,

*Hallowell, Me., Nov.* 1, 1848. M. C. RICHARDSON, M. D.

